# Leadership education for physicians—how it fits in their culture

**DOI:** 10.1007/s40037-019-0521-6

**Published:** 2019-06-03

**Authors:** Louis N. Pangaro

**Affiliations:** Department of Medicine (MED), F. Edward Hebert School of Medicine, Uniformed Services University, Bethesda, MD USA

The paper by Onyura et al. [1] is a warning to the medical education community that the role of physicians in the shaping of its future may be in jeopardy if it does not devote more time to developing leaders from within its ranks. Since medical education is closely bound to its medical healthcare system, the authors’ finding that fewer than 15% of leadership curricula dealt with leadership at the system level should be worrying.

The Onyura paper joins other recent reviews [2, 3] demonstrating that currently available leadership programs are not broad enough in their learning objectives nor robust enough in their pedagogy to meet the emerging challenges to the profession. Its main findings are that these programs focus on managerial issues (quality, finance, conflict resolution, etc.), leaving significant gaps in the content of leadership training; that such training is rarely interprofessional; and finally, that only a minority (20%) have sustained programs (a year in length) that include mentoring and experiences in leadership.

This Commentary will address the premises that physicians can and ought to be trained to be part of solving the complex problems of medical systems, problems thought to be so complex that solutions cannot be assumed to be permanent; hence the problems are ‘wicked’. Is medical education ready to develop leaders to deal with such problems?

The authors’ premise is that society will be better if physicians are involved in solving the complex, ‘wicked’ problems that face healthcare systems, e.g. an ageing population and emerging disease threats. What principles support this premise? Does the concept of professionalism as a promise of expertise and of duty [4] require that those directing patient care should have the competence needed for problems at the level of healthcare systems? And that they also have the required conviction to place the needs of the patient paramount among considerations of cost or profit?

How complicated or complex does an approach to leadership need to be to deal with wicked problems? All leadership theories (trait, behavioural, situational, path-goal, transformational, adaptive, etc. [5]). combine technical, task-oriented aspects (expertise) with those that are relational (duty). Of course, most professions combine these, and certainly medicine insists on both in great depth. This promise of both expertise and duty is in its simplicity an explicit refutation of the notion that it takes complexity to defeat complexity [6], or that one needs to be wicked (not evil, of course) to deal with the wicked problems.

Do physicians bring a unique and required appreciation of both the technical and humanistic dimensions of healthcare? While clinical diagnosis is mainly a cognitive process, shared decision-making with a patient moves into an area of choices and values, where single, best courses of action are not always available. This movement from understanding into action is the essence of medical education, moving the learner from the cognitive to ethical dimensions, and thus ready to bring expertise and duty to the solving of wicked problems.

Schein’s description of three cultures of management [7] can be used to locate the unique perspective that physicians bring to problem solving. Physicians are part of an ‘operator’ culture which solves problems on a case-by-case basis. The ‘executive culture’ of high level managers sets a vision for the organization, provides resources and protects the organization from outside threats. It also may align with an ‘engineering culture’ that sees the organization as a machine. The purpose of leadership education would be to move more physicians to the executive suite, and have all physicians engage in the process of interprofessional collaboration.

Is it fair to ask busy and beleaguered physicians to add yet another, formidable item to their curriculum to accept the challenges of health system leadership? How difficult will this be and how great a leap?

It has been said that ‘doctor means teacher’ and nearly all physicians accept the role, which is explicit in the Hippocratic oath. The etymologies of our common terms for the teaching—education and pedagogy—contain the first clue. ‘Education’ derives from the Latin words for ‘to lead out’ of (*e-ducere*) and ‘pedagogy’ from the Greek for ‘to lead a child’ (*ped-agogein*). They both contain the concept of leading from a dependent state (childhood) to an independent one, and this progress is the premise behind medical education, that we are fostering the independence of learners on their way toward unsupervised practice. And this is true whether for one student at a time or for a dozen of residents in a training program.

Some of our most impressive faculty development programs in teaching skills are predicated on the notion of physician as leader. In the Stanford Faculty Development Center teaching framework, we see the pillars of leadership as setting the learning climate, communicating expectations and giving feedback [8]. This entire conceptualization rises and converges in the independence of the learner (self-direction). Similarly, Steinert’s faculty development approach includes a change in attitude toward organizational contexts and changes in leadership behaviour [9].

How much of a conceptual change would the universal introduction of leadership curricula really be? We have heard program directors say that developing leaders is what they do—all the time. The needed changes are simply in the level in the ‘system’ in which we are engaged, not conceptual. In other words, physicians, who claim to understand nature (*physis* in Greek), can already engage problems at the granular level of molecules; at the level of organ function and homeostasis; at the level of decision-making with an individual patient; and at the level of getting a patient the right care in a clinical microsystem. At present medical education is committing itself to mastering ‘system science’ [10], i.e., working at the macro level. If the constant emergence of wicked problems requires complex adaptive leadership [7] and also the contemporary, shared responsibility advocated by Onyura, then surely the medical education community is ready for this challenge. Physicians can embrace complexity, and act with simplicity [11], and can commit themselves to a promise of expertise and a promise of duty in their leadership of patients, students and now also in healthcare collaboration.
